# Association Between Objectively Measured Sleep Duration and Symptoms of Psychiatric Disorders in Middle Childhood

**DOI:** 10.1001/jamanetworkopen.2019.18281

**Published:** 2019-12-27

**Authors:** Bror M. Ranum, Lars Wichstrøm, Ståle Pallesen, Jonas Falch-Madsen, Marte Halse, Silje Steinsbekk

**Affiliations:** 1 Department of Psychology, Norwegian University of Science and Technology (NTNU), Trondheim, Norway; 2 Department of Child and Adolescent Psychiatry, St Olavs Hospital, Trondheim, Norway; 3 NTNU Social Research, Human Development Department, Trondheim, Norway; 4 Department of Psychosocial Science, University of Bergen, Bergen, Norway; 5 Norwegian Competence Center for Sleep Disorders, Haukeland University Hospital, Bergen, Norway

## Abstract

**Importance:**

The long-term association between sleep duration and mental health in children is currently unknown.

**Objective:**

To investigate the prospective associations between sleep duration and symptoms of emotional and behavioral disorders at ages 6, 8, 10, and 12 years.

**Design, Setting, and Participants:**

This population-based cohort study obtained data from the Trondheim Early Secure Study in Trondheim, Norway. A representative, stratified random sample of children born between January 1, 2003, and December 31, 2004, were invited to participate. Participants were followed up biennially from age 4 years (2007-2008) to 12 years (2013-2014). Data analysis was conducted from January 2, 2019, to May 28, 2019.

**Main Outcomes and Measures:**

Sleep duration was assessed with 1 week of continuous use of a triaxial accelerometer. Symptoms of emotional (anxiety and depression) and behavioral (oppositional defiant, conduct, and attention-deficit/hyperactivity) disorders were measured by semistructured clinical interviews (using the Preschool Age Psychiatric Assessment and the Child and Adolescent Psychiatric Assessment) with parents (at all ages) and children (from age 8 years).

**Results:**

The analytical sample comprised 799 children (mean [SD] age at time point 2, 6.0 [0.2] years; 405 [50.7%] boys; and 771 [96.5%] Norwegian). Shorter sleep duration at age 6 years (β [unstandardized regression coefficient] = −0.44; 95% CI, −0.80 to −0.08; *P* = .02) and 8 years (β = −0.47; 95% CI, −0.83 to −0.11; *P* = .01) forecasted symptoms of emotional disorders 2 years later. Comparatively short sleep duration at age 8 years (β = −0.65; 95% CI, −1.22 to −0.08; *P* = .03) and 10 years (β = −0.58; 95% CI, −1.07 to −0.08; *P* = .02) was associated with symptoms of behavioral disorders 2 years later among boys but not among girls at age 8 years (β = −0.14; 95% CI,− 0.52 to 0.24; *P* = .48) or 10 years (β = −0.05; 95% CI, = −0.49 to 0.40; *P* = .84). These associations were statistically significant among boys compared with girls at age 8 years (Δχ^2^_1_ = 13.26; *P* < .001) and 10 years (Δχ^2^_1_ = 10.25; *P* = .001). Symptoms of psychiatric disorders did not forecast sleep duration at any age.

**Conclusions and Relevance:**

This study found an association between short sleep duration and increased risk of future occurrence of emotional disorder symptoms in both boys and girls and between reduced sleep and behavioral disorder symptoms in boys. These results suggest that improving sleep in children may help protect against the development of symptoms of common psychiatric disorders and may be advantageous in the treatment of such disorders.

## Key Points

**Question:**

What is the long-term and bidirectional association between sleep duration and symptoms of psychiatric disorders in school-aged children?

**Findings:**

In this population-based cohort study of 799 Norwegian children participating in the Trondheim Early Secure Study, when all time-invariant confounders and baseline levels of study variables were accounted for, short sleep duration was prospectively associated with symptoms of psychiatric disorders at younger but not older ages. No evidence was found for the opposite direction of association.

**Meaning:**

This study found that short sleep duration was associated with the development of symptoms of psychiatric disorders, but symptoms of psychiatric disorders were not associated with reduced sleep.

## Teaser

This population-based cohort study examines whether sleep duration is associated with the development of emotional and behavioral problems in school-aged children in Norway.

## Introduction

Short sleep duration plays a role in the development of both behavioral and emotional problems in children. Reduced sleep seems to impair executive functions,^1^ such as impulse control and cognitive flexibility. Impaired executive functioning, in turn, is associated with behavioral problems^2^ that possibly manifest as increased anger,^3^ impulsivity, and decreased cognitive flexibility. Curtailed sleep has been found to diminish positive emotions,^4^ which may be a factor in higher risk of emotional disorders given that having positive emotions run counter to simultaneously feeling depressed or anxious. Positive emotions, such as joy, may ward off mental illness by eliciting playful behavior, which builds good social relationships and physical capabilities.^5^ In addition, impaired emotion regulation resulting from sleep loss may increase the risk of developing emotional disorders by increasing the use of maladaptive emotion regulation strategies,^5^ such as rumination, suppression, and avoidance.^6^

Longitudinal observational studies based on subjective, self-reported sleep duration have found that loss of sleep is prospectively associated with mental health problems, such as anxiety, depression, and poor emotion regulation.^7^ However, subjective reports of sleep duration are often tainted by sleep misperception^8^ and may, together with common method bias^9^ and modest associations with objectively measured sleep,^10^ obscure the true association between sleep and psychiatric disorders. Objective measures of sleep are often applied in experimental research, which has consistently found that curtailed sleep is associated with diminished mental health abilities in children, such as emotion regulation,^4^ cognitition,^11^ and attention.^12^ However, longitudinal studies with objective measures of sleep are needed to ascertain whether reduced sleep forecasts mental health problems beyond the immediate effects. Given the lack of such data, the aim of the present cohort study is to test this proposition.

A strong case has also been made to examine the opposite or reverse direction of association: mental health being associated with sleep.^13^ It has been theorized that emotional problems can exacerbate sleep problems through rumination and worry, which require high levels of arousal and vigilance that are, in turn, associated with longer sleep latency, more nocturnal awakenings, or earlier termination of sleep in the morning.^13^ Behavioral disorders, such as attention-deficit/hyperactivity disorder, may be associated with sleep-onset difficulties such as hyperarousal, bedtime resistance, and night awakenings from body movements,^14^ whereas oppositional defiant disorder and conduct disorder typically are associated with loss of sleep owing to bedtime resistance.^15^

Although many clinical studies have shown that children with mental health problems get less sleep compared with those without such problems,^16^ the direction of association cannot be inferred, and results from clinical samples may not generalize to children with standard development. To our knowledge, only 3 previous studies have investigated whether objectively measured sleep duration forecasts later occurrence of mental health problems among prepubertal school-aged children. One study^17^ found no evidence of higher risk of anxiety and depression in children aged 6 to 12 years who slept 7.5 or fewer hours as measured by polysomnography compared with children who slept 9 or more hours at follow-up 5 years later. Corroborating these results were findings of a second study^18^ that sleep duration measured by actigraphy in children at age 9 years did not forecast internalizing or externalizing symptoms at age 10 years. However, short sleep duration measured by actigraphy at age 10 years forecasted symptoms of behavioral and emotional disorders at age 13 years according to a third study.^19^ Only 1 study^19^ has investigated the opposite direction of association (whether poor mental health influences sleep duration), reporting that depression forecasted shorter sleep duration from age 8 to 10 years but not from age 10 to 12 years.

Although these studies represent important steps toward identifying potential long-term reciprocal associations between sleep duration and mental health in children, several methodological limitations in these studies may preclude firm conclusions about the direction and magnitude of association. First, the studies may have been underpowered to detect small to moderate associations. Second, findings from self-administered questionnaires used to assess mental health problems may show only modest associations with results obtained by clinical interviews.^20^ Clinical interviews allow probing for additional information concerning the intensity, duration, and onset of a psychiatric symptom to ascertain its actual presence.^21^ Third, a range of potential confounding variables (eg, genetics,^22^ marital conflict,^23^ and parenting^24^) may be factors in both sleep duration and mental health, producing spurious associations that cannot be easily ruled out in observational research. Many such confounders are time invariant (eg, genes) or have a large time-invariant component (ie, do not substantially change over time, such as stable parenting characteristics). Time-invariant confounders can be adjusted by applying a dynamic panel model approach,^25^ which heretofore has not been used in childhood sleep research, to our knowledge. In addition, the direction of association between sleep and mental health might be contingent on individual characteristics, such as sex. Although scarce, evidence has shown that sleep restriction affects men and women differently.^26^ For example, men become more willing to take risks,^27^ whereas women’s emotional recognition is more negatively affected,^28^ and the biological stress response in female adolescents is more negatively affected compared with male adolescents.^29^ Furthermore, cumulative sleep deprivation has been associated with higher risk of depression in female but not in male study participants^30^; among adolescents with emotional difficulties, female, but not male, teenagers have been found at risk for sleep problems.^31^ Thus, the association between reduced sleep and mental health may be contingent on sex.

This cohort study, taking the methodological limitations of previous research into account, investigated the direction of association, including sex differences, between objectively measured sleep duration and symptoms of emotional and behavioral disorders as defined by the *Diagnostic and Statistical Manual of Mental Disorders* (Fourth Edition, Text Revision) (*DSM-IV-TR*).^32^ With a multiwave longitudinal design, this study examined a representative middle childhood population and statistically adjusted for all unmeasured time-invariant confounders.

## Methods

### Participants and Procedure

Data were derived from the Trondheim Early Secure Study (TESS), an ongoing community study of the mental, psychosocial, and behavioral health of children and youth in Trondheim, Norway.^33^ All children born in Trondheim receive an appointment for a routine well-child care visit along with an invitation, sent to their parents, to participate in TESS. A screening questionnaire for emotional and behavioral problems—the Strengths and Difficulties Questionnaire (SDQ), version 4-16—is also sent to the parents.^34^ When children and their parents arrive at their community clinic for the appointment, the health nurse at the clinic informs the parents about the TESS and collects written informed consent forms from those who want to participate. The present analysis focused on the cohort of children born between January 1, 2003, and December 31, 2004. All procedures used in TESS were reviewed and approved by the Regional Committee for Medical and Health Research Ethics Mid-Norway. A flowchart of recruitment and attrition of the TESS main sample is depicted in the Figure. The present cohort study received approval from the Regional Committee for Medical and Health Research Ethics Mid-Norway. We followed the Strengthening the Reporting of Observational Studies in Epidemiology (STROBE) reporting guideline.

**Figure.**
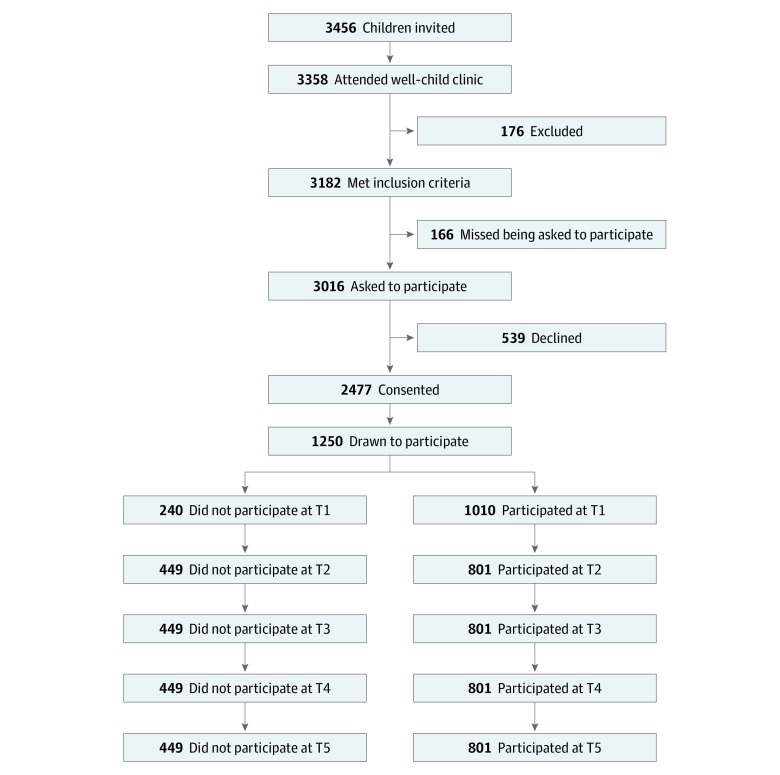
Flowchart of the Recruitment and Follow-up The number of participants at various assessment points was based on the number of participants invited to participate (n = 1250) minus those who did not participate at the respective measurement time points (ie, time 1 [T1] through time 5 [T5]).

Participants were followed up biennially from age 4 years (2007-2008) to 12 years (2013-2014). To increase statistical power, children with emotional or behavioral problems were oversampled; therefore, participants were allocated to 4 strata according to their SDQ scores (cutoff scores: 0-4, 5-8, 9-11, and 12-40 points). The probability of selection increased with higher SDQ scores: 0.37 with 0-4 points, 0.48 with 5-8 points, 0.70 with 9-11 points, and 0.89 with 12-40 points. The number of participants drawn from each stratum totaled 1095 from the SDQ 0-4 group, 731 from the SDQ 5-8 group, 455 from the SDQ 9-11 group, and 194 from the SDQ 12-40 group. The number of participants actually interviewed at time point 1 (age 4 years) for each stratum was 316 from the SDQ 0-4 group, 292 from the SDQ 5-8 group, 249 from the SDQ 9-11 group, and 138 from the SDQ 12-40 group.

Population weights were applied in the analyses to provide corrected population estimates. For time point 1, the children randomly selected for inclusion had a mean (SD) age of 4.7 (0.3) years at baseline, and 496 (49.1%) were boys. Accelerometer assessment of sleep was implemented from age 6 years onward; therefore, data from time point 1 (age 4 years) were not included in the present analyses. Participants with valid accelerometer data on at least 1 occasion composed the analytical sample (n = 799) and were considered a subsample of the main TESS sample (eTable 1 in the Supplement). Accelerometer measurements and interviews occurred biennially at age 6 years (n = 687; mean [SD] age, 6.0 [0.17] years), age 8 years (n = 619; mean [SD] age, 8.8 [0.24] years), age 10 years (n = 618; mean [SD] age, 10.5 [0.17] years), and age 12 years (n = 558; mean [SD] age, 12.5 [0.14] years).

### Measures

#### Sleep Duration

Sleep duration was assessed by a triaxial accelerometer (ActiGraph GT3X; Manufacturing Technology Inc). The accelerometer is a small, hip-strapped device that measures acceleration in 3 dimensions. Children were instructed to wear the accelerometer for 7 consecutive days, 24 hours per day, only taking it off when bathing or showering. Bedtime and rise time were manually scored by inspecting each individual actogram. Human visual scoring has been found to have superior correlation with polysomnography compared with autodetection by computer^35^ and is closely aligned with sleep diary entries, with a miniscule (0.7 minute) difference in sleep duration.^36^ Sleep duration (in minutes; scored as asleep between bedtime and rise time, excluding minutes awake) was derived using the Sadeh sleep algorithm^37^ with 60-second epochs (time blocks).^38^ The Sadeh algorithm scores an epoch as asleep or awake depending on activity during the epoch, considering the activity in the previous and subsequent 5 minutes. Sleep duration in hours was averaged across the week of measurement. Only nocturnal sleep was considered.

#### Symptoms of Psychiatric Disorders

The Preschool Age Psychiatric Assessment (PAPA), a semistructured psychiatric interview of parents, was used to assess psychiatric symptoms in children at age 6 years. At 3 consecutive follow-ups (at ages 8, 10, and 12 years), the Child and Adolescent Psychiatric Assessment (CAPA) was administered. The PAPA and CAPA instruments comprise structured protocols with required and optional follow-up questions for identifying *DSM-IV-TR*—defined symptoms. With CAPA, the parent and child were interviewed separately, and a symptom was considered to be present if it was reported by either the parent or child. Symptoms were categorized as emotional (ie, separation anxiety, generalized anxiety disorder, social phobia, specific phobia, major depressive disorder, and dysthymia) or behavioral (ie, attention-deficit/hyperactivity disorder, oppositional defiant disorder, and conduct disorder). Blinded raters recoded 89 PAPA and 187 CAPA interviews. Multivariate interrater reliabilities^39^ (intraclass correlation coefficients) between multiple pairs of blinded raters for emotional disorders were 0.93 for PAPA and 0.82 for CAPA, whereas reliabilities for behavioral disorders were 0.97 for PAPA and 0.83 for CAPA.

### Statistical Analysis

All statistical analyses were performed in Mplus, version 7.4.^40^ Missing data were handled by applying full information maximum likelihood.^41^ Because we oversampled children with high SDQ scores, data were weighted according to the number of children in the stratum in the population divided by the number of participating children in that specific stratum. A robust maximum likelihood estimator was used,^42^ which did not presuppose multivariate normality. To assess the prospective associations between sleep duration and symptoms of psychiatric disorders, we applied fixed and hybrid dynamic panel modeling (eFigure, eEquation, eAppendix 1, and eAppendix 2 in the Supplement).^43,44^ In such a fixed-effects analysis, sleep duration, symptoms of emotional disorders, and symptoms of behavioral disorders at time point *t* were regressed on these measures at *t – *1. Error terms at the same time point were allowed to correlate. Three latent factors, with 1 for each measure (sleep duration, symptoms of behavioral disorders, and symptoms of emotional disorders), were created, and measures were loaded at ages 8, 10, and 12 years. These time-invariant factors were allowed to correlate with each other and with the initial measures (eg, sleep duration, symptoms of behavioral disorders, and symptoms of emotional disorders at age 6 years), which were considered exogenous. Hence, the prospective association between 1 conditional independent variable (eg, sleep duration) at *t* and 1 conditional dependent variable (eg, symptoms of emotional disorders) at *t* + 1 was estimated, adjusting for time-invariant factors. Because of their exclusive reliance on within-person variance, such fixed-effects models had limited statistical power.

In contrast, hybrid models (ie, models in which negligible correlations between covariates and time-invariant latent variables were set to 0) retained the fixed-effects advantage of adjusting for time-invariant factors while being more parsimonious and preserving statistical power.^45^ We therefore tested whether a hybrid model would fit data equally or better than a fixed-effects model using the Satorra-Bentler scaled χ^2^ test,^46^ which is a functional equivalent to the Hausman test.^43^ To assess sex differences in long-term associations between sleep duration and symptoms of psychiatric disorders, we ran the hybrid model grouped by sex. Then, we compared the hybrid model grouped by sex (sex-specific coefficients estimated freely) with a hybrid model grouped by sex with sex-specific coefficients constrained to be equal. The Satorra-Bentler–scaled χ^2^ test was used to identify which model best fit the data. Associations with 2-sided *P* ≤ .05 were considered significant. Data analysis was conducted from January 2, 2019, to May 28, 2019.

## Results

Overall, 799 participants (mean [SD] age at time point 2, 6.0 [0.2] years; 405 boys [50.7%] and 395 girls [49.3%]; and 771 [96.5%] Norwegian) were included in the analysis. All 799 children had usable sleep data on at least 1 measurement wave and thus constituted the analytical sample. The dropout rate after consenting to participate did not vary by SDQ score (*t*_1_ = 0.17; *P* = .86) or sex (Cramer *v*^2^_1_ = 1.02; *P* = .31) at time point 1. Neither sleep duration nor symptoms of behavioral or emotional disorders were associated with attrition at any time point. Table 1 displays the characteristics of the analytic sample at time point 2 (N = 687; mean [SD] age, 6.0 [0.17] years; 348 girls [50.6%]).

**Table 1.** Unweighted Sample Characteristics of Participants With Valid Accelerometer Data at Time Point 2^a^

Variable	No. (%)
Sex of child	
Male	335 (48.8)
Female	348 (50.6)
Missing data	4 (0.6)
Sex of parent informant	
Male	120 (17.5)
Female	548 (79.8)
Missing data	19 (2.8)
Ethnic origin of biological mother	
Norwegian	601 (87.5)
Western countries^b^	23 (3.4)
Other countries	27 (3.9)
Missing data	36 (5.2)
Ethnic origin of biological father	
Norwegian	586 (85.3)
Western countries^b^	40 (5.8)
Other countries	21 (3.1)
Missing data	40 (5.8)
Biological parents’ marital status	
Married	392 (57.1)
Cohabitating	179 (26.1)
Divorced/separated	94 (13.7)
Other	11 (1.6)
Missing data	11 (1.6)
Informant parents’ socioeconomic status	
Leaders	44 (6.4)
Higher professionals	165 (24)
Lower professionals	255 (37.1)
Skilled workers	140 (20.4)
Farmers or fishermen	1 (0.2)
Unskilled workers	19 (2.8)
Missing data	63 (9.2)
Household gross annual income, NOK [USD]	
0 to 225 000 [0 to 26 500]	17 (2.5)
>225 000 to 525 000 [>26 500 to 62 000]	82 (11.9)
>525 000 to 900 000 [>62 000 to 106 000]	308 (44.8)
>900 000 [>106 000]	270 (39.3)
Missing data	10 (1.5)

Abbreviations: NOK, Norwegian krone; USD, US dollar.

^a^ The analytical sample (N = 687) was aged 6 years.

^b^ Western countries include Western Europe, United States, Canada, New Zealand, and the Nordic countries except Norway.

Detailed descriptive statistics of study variables are presented in Table 2 (eTable 2 in the Supplement shows missing values). Bivariate correlations between study variables are displayed in Table 3.

**Table 2.** Sample Size

Variable	Participants, No.	Mean (SD)	Median (20th-80th Percentile) [Range]
Short sleep duration, h			
Age 6 y	685	9.66 (0.58)	9.70 (9.21-10.13) [7.06-11.38]
Age 8 y	618	9.26 (0.58)	9.25 (8.74-9.70) [6.59-11.14]
Age 10 y	617	9.01 (0.57)	9.02 (8.53-9.49) [7.03-10.00]
Age 12 y	557	8.69 (0.61)	8.69 (8.17-9.16) [6.82-10.63]
Symptoms of emotional disorders, No.			
Age 6 y	756	1.77 (2.48)	1.00 (0-4.00) [0-15.00]
Age 8 y	694	1.80 (2.35)	1.00 (0-4.00) [0-20.00]
Age 10 y	697	2.34 (2.83)	2.00 (0-4.00) [0-23.00]
Age 12 y	557	2.40 (3.21)	2.00 (0-5.00) [0-21.00]
Symptoms of behavioral disorders, No.			
Age 6 y	756	2.45 (3.09)	2.00 (0-5.00) [0-23.00]
Age 8 y	682	2.59 (3.44)	2.00 (0-5.00) [0-23.00]
Age 10 y	694	2.63 (3.40)	2.00 (0-5.00) [0-25.00]
Age 12 y	651	2.23 (3.21)	1.00 (0-4.00) [0-17.00]

**Table 3.** Correlations of Study Variables^a^

Variable	Short Sleep Duration, Age 8 y	*P* Value	Short Sleep Duration, Age 10 y	*P* Value	Short Sleep Duration, Age 12 y	*P* Value	Symptoms of Emotional Disorders, Age 6 y	*P* Value	Symptoms of Emotional Disorders, Age 8 y	*P* Value	Symptoms of Emotional Disorders, Age 10 y	*P* Value	Symptoms of Emotional Disorders, Age 12 y	*P* Value	Symptoms of Behavioral Disorders, Age 6 y	*P* Value	Symptoms of Behavioral Disorders, Age 8 y	*P* Value	Symptoms of Behavioral Disorders, Age 10 y	*P* Value	Symptoms of Behavioral Disorders, Age 12 y	*P* Value
Short sleep duration, age 6 y	0.38	<.001	0.38	<.001	0.29	<.001	−0.08	.05	−0.08	.10	0.04	.28	0.07	.09	−0.07	.06	−0.05	.23	−0.06	.22	0.05	.22
Short sleep duration, age 8 y	NA	NA	0.45	<.001	0.36	<.001	−0.11	.008	−0.12	.01	−0.11	.008	−0.07	.10	−0.12	.003	−0.10	.01	−0.11	.02	−0.06	.13
Short sleep duration, age 10 y	NA	NA	NA	NA	0.44	<.001	–0.12	.002	–0.11	.006	–0.11	.01	−0.03	.59	−0.11	.01	−0.03	.45	−0.11	.006	−0.08	.06
Short sleep duration, age 12 y	NA	NA	NA	NA	NA	NA	–0.06	.30	–0.07	.13	–0.02	.74	−0.03	.51	−0.11	.02	−0.01	.83	−0.01	.03	0.01	.89
Symptoms of emotional disorders, age 6 y	NA	NA	NA	NA	NA	NA	NA	NA	0.33	<.001	0.25	<.001	0.23	<.001	0.50	<.001	0.33	<.001	0.29	<.001	0.29	<.001
Symptoms of emotional disorders, age 8 y	NA	NA	NA	NA	NA	NA	NA	NA	NA	NA	0.35	<.001	0.37	<.001	0.35	<.001	0.47	<.001	0.36	<.001	0.39	<.001
Symptoms of emotional disorders, age 10 y	NA	NA	NA	NA	NA	NA	NA	NA	NA	NA	NA	NA	0.45	<.001	0.27	<.001	0.32	<.001	0.41	<.001	0.39	<.001
Symptoms of emotional disorders, age 12 y	NA	NA	NA	NA	NA	NA	NA	NA	NA	NA	NA	NA	NA	NA	0.22	<.001	0.24	<.001	0.33	<.001	0.52	<.001
Symptoms of behavioral disorders, age 6 y	NA	NA	NA	NA	NA	NA	NA	NA	NA	NA	NA	NA	NA	NA	NA	NA	0.59	<.001	0.52	<.001	0.46	<.001
Symptoms of behavioral disorders, age 8 y	NA	NA	NA	NA	NA	NA	NA	NA	NA	NA	NA	NA	NA	NA	NA	NA	NA	NA	0.66	<.001	0.55	<.001
Symptoms of behavioral disorders, age 10 y	NA	NA	NA	NA	NA	NA	NA	NA	NA	NA	NA	NA	NA	NA	NA	NA	NA	NA	NA	NA	0.66	<.001
Symptoms of behavioral disorders, age 12 y	NA	NA	NA	NA	NA	NA	NA	NA	NA	NA	NA	NA	NA	NA	NA	NA	NA	NA	NA	NA	NA	NA

Abbreviation: NA, not applicable.

^a^ Values represents Pearson correlations with accompanying *P* values.

The hybrid model had a good fit with the data (eTable 3 in the Supplement), and model fit was not statistically significantly different (Δχ^2^_4_ = 5.24; *P* = .26) from the fixed-effects model (eTable 3 in the Supplement). Because we expected low to moderate associations between sleep duration and symptoms of psychiatric disorders and because the prevalence of these symptoms was relatively low, we preferred the hybrid model; it provided more statistical power while retaining most of the fixed-effects advantage (eAppendix 3 in the Supplement). As shown in Table 4, short sleep duration at age 6 years forecasted more symptoms of emotional disorders but not behavioral disorders at age 8 years (β = −0.44 [95% CI, −0.80 to −0.08], *P* = .02 for emotional disorders; β = −0.35 [95% CI, −0.73 to −0.05], *P* = .08 for behavioral disorders), whereas short sleep duration at age 8 years forecasted both emotional and behavioral symptoms at age 10 years (β = −0.47 [95% CI, −0.83 to −0.11], *P* = .01 for emotional disorders; β = −0.49 [95% CI, −0.90 to −0.07]; *P* = .02 for behavioral disorders). Short sleep duration at age 10 years did not forecast symptoms at 12 years of age. Moreover, no evidence of a reverse direction of association was found; neither symptoms of emotional disorders nor symptoms of behavioral disorders forecasted sleep duration at any *t* + 1 time point (see eTable 4 in the Supplement for all regression coefficients in the model).

**Table 4.** Unstandardized Regression Coefficients (β) From the Hybrid Model Testing Long-term Associahtions Between Sleep Duration and Symptoms of Psychiatric Disorders

Association Between Variables	β (95% CI)	*P* Value
Symptoms of emotional disorders at age 8 y, regressed on sleep duration at age 6 y	−0.44 (−0.80 to −0.08)	.02
Symptoms of emotional disorders at age 10 y, regressed on sleep duration at age 8 y	−0.47 (−0.83 to −0.11)	.01
Symptoms of emotional disorders at age 12 y, regressed on sleep duration at age 10 y	0.05 (−0.40 to −0.50)	.83
Symptoms of behavioral disorders at age 8 y, regressed on sleep duration at age 6 y	−0.35 (−0.73 to −0.05)	.08
Symptoms of behavioral disorders at age 10 y, regressed on sleep duration at age 8 y	−0.49 (−0.90 to −0.07)	.02
Symptoms of behavioral disorders at age 12 y, regressed on sleep duration at age 10 y	−0.33 (−0.69 to −0.04)	.08
Short sleep duration at age 8 y, regressed on symptoms of emotional disorders at age 6 y	0.00 (−0.03 to −0.02)	.73
Short sleep duration at age 8 y, regressed on symptoms of behavioral disorders at age 6 y	0.00 (−0.02 to −0.02)	.90
Short sleep duration at age 10 y, regressed on symptoms of emotional disorders at age 8 y	−0.02 (−0.04 to −0.01)	.14
Short sleep duration at age 10 y, regressed on symptoms of behavioral disorders at age 8 y	0.01 (−0.01 to −0.02)	.38
Short sleep duration at age 12 y, regressed on symptoms of emotional disorders at age 10 y	0.00 (−0.03 to −0.02)	.81
Short sleep duration at age 12 y, regressed on symptoms of behavioral disorders at age 10 y	0.00 (−0.01 to −0.02)	.58

Baseline levels of sleep duration and symptoms were adjusted for in the model.

As for the magnitude of associations, a child with half an hour shorter sleep duration at age 6 years tended to have 0.22 more symptoms of emotional disorders at age 8 years, assuming all other factors were constant. A child with half an hour shorter sleep duration at age 8 years tended to have 0.24 more symptoms of emotional disorders and 0.25 more symptoms of behavioral disorders at age 10 years, assuming all other factors were constant.

Sex-specific analyses revealed that the path from short sleep durat−on at age 8 years to symptoms of behavioral disorders at age 10 years was evident among boys but not girls (boys: β [unstandardized regression coefficient] = −0.65; 95% CI, −1.22 to −0.08; *P* = .03 vs girls: β = −0.14; 95% CI, −0.52 to 0.24; *P* = .48), and this sex-specific association was significant (Δχ^2^_1_ = 13.26; *P* < .001). Reduced sleep at age 10 years forecasted more symptoms of behavioral disorders 2 years later among boys (β = −0.58; 95% CI, −1.07 to −0.08; *P* = .02) but not girls (β = −0.05; 95% CI, −0.49 to 0.40; *P* = .84), and this sex difference was also significant (Δχ^2^_1_ = 10.25; *P* = .001). The association between emotional disorders and short sleep duration 2 years earlier did not vary by sex (age 6-8 years: Δχ^2^_1_ = 0.25, *P* = .62; age 8-10 years: Δχ^2^_1_ = 0.14, *P* = .71; and age 10-12 years: Δχ^2^_1_ = 0.06, *P* = .81).

## Discussion

We examined whether short sleep duration was associated with future symptoms of emotional and behavioral disorders in middle childhood and whether symptoms of such disorders were associated with future reduced sleep. A large community sample of Norwegian children was followed up biennially from age 6 years until 12 years for measurement of sleep duration using an accelerometer and clinical interviews. We adjusted for previous levels of sleep duration and psychiatric symptoms as well as for unmeasured time-invariant confounders. We found partial evidence for a long-term association of short sleep duration with later occurrence of symptoms of emotional disorders and behavioral disorders in male participants, but no evidence for the opposite direction of association was found.

The findings in this cohort study are at odds with those of previous studies that reported no association between sleep duration and mental health in children.^17,18,19^ The associations found in this study were modest, and the CIs for the associations between sleep duration and later symptoms of psychiatric disorders were relatively large; thus, previous investigations may have been underpowered to detect associations of this magnitude. Moreover, studies that used respondent-based assessments of mental health tended to report considerably higher stability of mental health problems^47^ compared with studies that used interviewer-based assessments.^48^ Such results suggested the presence of a strong common rater bias in past studies, implying that less variation in sleep had to be explained, which may have further exacerbated power problems in modest-sized studies.

This study’s findings also suggest that short sleep duration may be an antecedent of poor mental health, and these associations were evident when all time-invariant confounders were accounted for. Although short nocturnal sleep duration in children is often followed by longer sleep on subsequent nights,^49^ such compensation probably does not fully remediate the negative consequences of insufficient sleep.^50^ Hence, the negative consequences of short sleep duration might linger for days; if sleep loss repeatedly occurs, over time, it may possibly direct developmental patterns into several unhealthy pathways.

First, sleep reduction may be associated with misperceptions of social cues, which may affect emotional expression and may, in turn, lead to problems in social interactions. In adults, 1 night of total sleep deprivation has been shown to blunt the ability to recognize angry and happy faces^28^ and the ability to discriminate between socially threatening and neutral faces.^51^ If this consequence holds true for children and occurs even with mild sleep loss, a developmental trajectory toward poor social skills is a possible outcome, which may increase the risk of peer rejection, aggression, bullying, and perception of less belongingness, all of which are associated with mental health problems.^52^ Second, short sleep duration may bias neurocognitive systems, rendering individuals more likely to develop mental health problems. This consequence may occur because sleep deprivation can impose a negative remembering bias in that sleep-deprived individuals tend to remember negative experiences and forget positive ones.^53^ Furthermore, an increase in negative mood, which has been observed in sleep-deprived adolescents,^54^ might be associated with difficulties in regulating emotion as well as intensifying and prolonging depressive mood in children.^13^ Sleep deprivation may also be associated with heightened stress reactivity within the hypothalamic-pituitary-adrenal axis,^55^ increasing the risk of psychopathological disorder.^56^ Third, lack of sleep in children may be associated with greater parental stress and fatigue,^57^ increasing the risk of maladaptive parenting strategies^58^ that, in turn, may contribute to the development of mental health problems.^59^

We found a sex-specific association between short sleep duration and symptoms of behavioral disorders in boys but not in girls. Previous research on adults also found a sex-specific outcome of sleep restriction (eg, male participants became more risk prone and female participants became more risk averse after sleep restrictions).^27^ A potential explanation for this finding is that parents may react differently to outcomes of short sleep duration (eg, attention problems, moodiness) in boys than in girls. Specifically, boys are more prone to receive or evoke harsh parenting,^60^ which can increase behavioral problems through coercive cycles,^61^ thereby adding to the sex-specific association with short sleep duration. We did not find a sex-specific association between risk of developing symptoms of emotional disorders and short sleep duration, a finding that is not in line with previous findings that reduced sleep is associated with emotional disorders in female adolescents.^30^ The female preponderance in emotional disorders typically emerges in early adolescence.^62^ Hence, the sex-specific, detrimental association between short sleep duration and emotional disorders in girls may not emerge until early adolescence, a period that was not included in the present study.

A causal relationship between short sleep duration and psychiatric symptoms cannot be inferred from this study. However, the findings suggest that mental health interventions for children could include improving their sleep duration, although treatment and prevention studies are needed to substantiate this recommendation.

### Strengths and Limitations

Although the present cohort study has many strengths, including a large population sample with repeated follow-ups, use of objectively measured sleep, diagnostic interviews, and a strong statistical approach, the findings should be considered in the context of its limitations. The prevalence of psychiatric disorders is relatively low in Norway^63^; thus, generalization to other countries must be made with caution. Likewise, children in the present study reported considerably longer sleep duration compared with children in several previous studies.^17,18,19,64^ Therefore, lower rates of short sleep duration may have diminished the reported associations. Because of the 2-year time span between assessments, short-term and transient outcomes may have gone undetected. Sleep duration was assessed with accelerometers, but these devices register only body movements, precluding the analysis of sleep architecture or sleep quality. Parental psychiatric disorder has been found to be associated with child psychiatric disorder,^65^ and that association may have confounded the results of the present study. However, we adjusted for time-invariant factors, such as stable mental health conditions in parents. Even so, some mental health conditions, such as depression, may wax and wane, and this reality could have affected offspring sleep and their future symptoms of psychiatric disorders. Although we adjusted for time-invariant confounders, this study had an observational design in which unobserved time-variant cofounders, such as negative life events, parental psychiatric disorder, transient episodes of bullying, change of school or other transitions, or changes in a child’s environment, could potentially have implications for the results and thus preclude causal conclusions.

## Conclusions

Findings from this cohort study suggest that short sleep duration at ages 6 and 8 years may forecast the occurrence of symptoms of emotional disorders 2 years later and that short sleep duration at ages 8 and 10 years may forecast symptoms of behavioral disorders in boys. No evidence was found for the opposite direction of association. Although we could not infer a causal relationship between reduced sleep and psychiatric symptoms, we believe the findings suggest that targeting sleep duration could be advantageous for mental health interventions. Treatment and prevention studies are needed to substantiate this suggestion.

## Notes

Supplement. **eFigure.** The Dynamic Panel Model: Cross-Lagged Part (Normal Font) and Time-Invariant Factor Part (Bold Font) **eEquation.** Regression Equation for the Dynamic Panel Model **eTable 1.** Participation Patterns for Children With Valid Sleep Measures **eTable 2.** Missing Values on Study Variables **eTable 3.** Results of Model Fitting Procedure **eTable 4.** All Unstandardized Regression Coefficients (B) From the Hybrid Model Testing Longitudinal Associations Between Sleep Duration and Symptoms of Psychiatric Disorders **eAppendix 1.** Mplus Code for a Hybrid-, Fixed-, and Random-Effects Dynamic Panel Model Testing Longitudinal Associations Between Sleep Duration and Symptoms of Psychiatric Disorders **eAppendix 2.** The Dynamic Panel Model (Random, Fixed, and Hybrid Effects Models): General Issues **eAppendix 3.** Fitting of the Models **eReferences.**
