# Supplementary material for: Association Between Objectively Measured Sleep Duration and Symptoms of Psychiatric Disorders in Middle Childhood
Source: JAMA Netw Open. 2019 Dec 27;2(12):e1918281. doi: 10.1001/jamanetworkopen.2019.18281 (PMC6991225; doi:10.1001/jamanetworkopen.2019.18281)
Supplement: Supplement. — eFigure. The Dynamic Panel Model: Cross-Lagged Part (Normal Font) and Time-Invariant Factor Part (Bold Font) eEquation. Regression Equation for the Dynamic Panel Model eTable 1. Participation Patterns for Children With Valid Sleep Measures eTable 2. Missing Values on Study Variables eTable 3. Results of Model Fitting Procedure eTable 4. All Unstandardized Regression Coefficients (B) From the Hybrid Model Testing Longitudinal Associations Between Sleep Duration and Symptoms of Psychiatric Disorders eAppendix 1. Mplus Code for a Hybrid-, Fixed-, and Random-Effects Dynamic Panel Model Testing Longitudinal Associations Between Sleep Duration and Symptoms of Psychiatric Disorders eAppendix 2. The Dynamic Panel Model (Random, Fixed, and Hybrid Effects Models): General Issues eAppendix 3. Fitting of the Models eReferences. [file jamanetwopen-2-e1918281-s001.pdf]

Supplementary Online Content

Ranum BM, Wichstrøm L, Pallesen S, Falch-Madsen J, Halse M, Steinsbekk S. Association between objectively measured sleep duration and symptoms of psychiatric disorders in middle childhood. *JAMA Netw Open.* 2019;2(12):e1918281. doi:10.1001/jamanetworkopen.2019.18281

eFigure. The Dynamic Panel Model: Cross-Lagged Part (Normal Font) and Time-Invariant Factor Part (Bold Font)

eEquation. The Regression Equation for the Dynamic Panel Model

eTable 1. Participation Patterns for Children With Valid Sleep Measures

eTable 2. Missing Values on Study Variables

eTable 3. Results of Model Fitting Procedure

eTable 4. All Unstandardized Regression Coefficients (B) From the Hybrid Model Testing Longitudinal Associations Between Sleep Duration and Symptoms of Psychiatric Disorders

eAppendix 1. Mplus Code for a Hybrid, Fixed, and Random Effects Dynamic Panel Model Testing Longitudinal Associations Between Sleep Duration and Symptoms of Psychiatric Disorders

eAppendix 2. The Dynamic Panel Model (Random, Fixed, and Hybrid Effects Models): General Issues

eAppendix 3. Fitting of the Models

eReferences.

This supplementary material has been provided by the authors to give readers additional information about their work.

© 2019 Ranum BM et al. *JAMA Network Open.*

eFigure. The Dynamic Panel Model: Cross-Lagged Part (Normal Font) and Time-Invariant Factor Part (Bold Font)

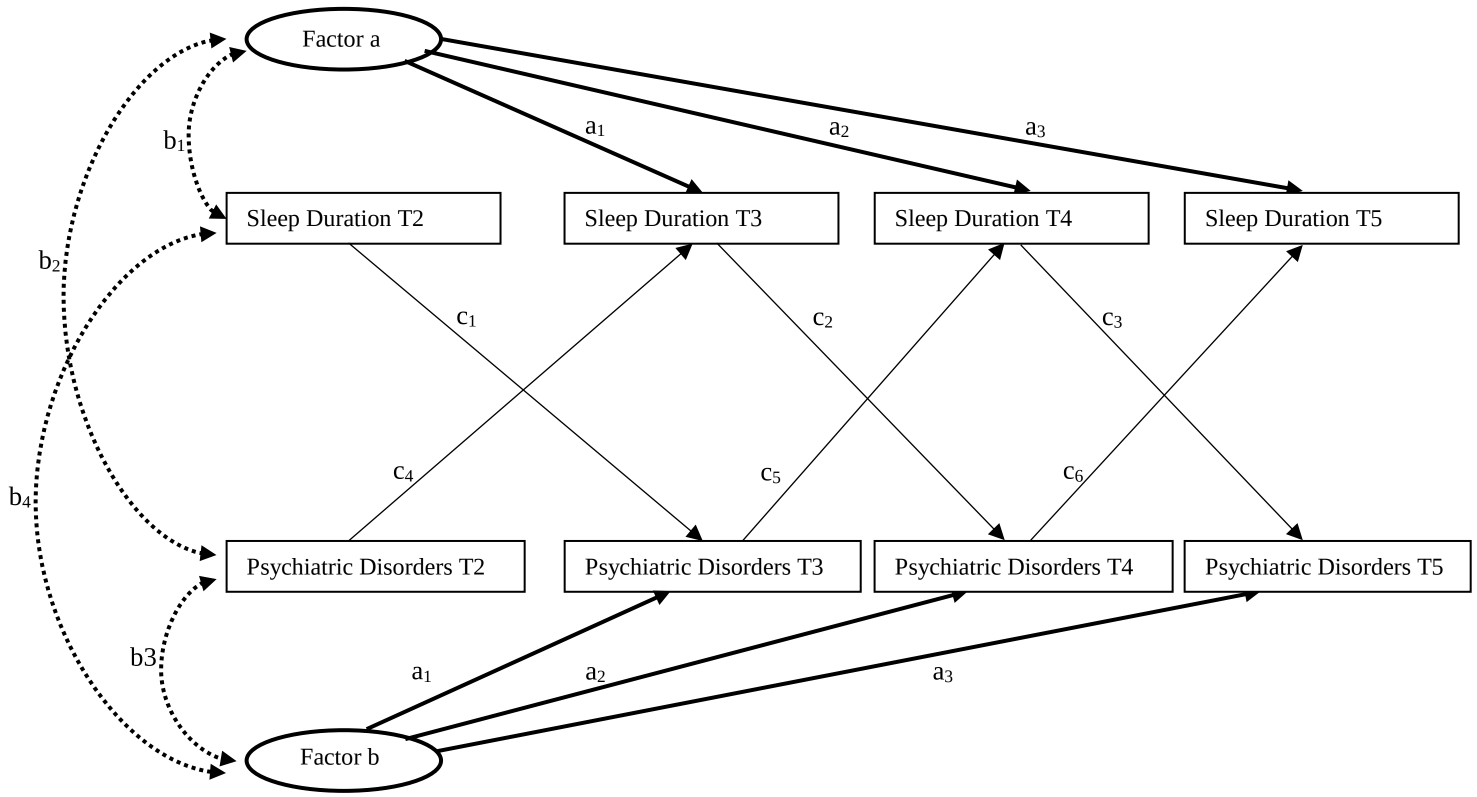

Note: Presentation of the analytical model tested. T2: Age 6; T3: Age 8; T4: Age 10; T5: Age 12. Note that the model is abbreviated for illustrative purposes. The model consists of a time-invariant factor for sleep duration (measured at T2, T3, T4, and T5), a time-invariant factor for symptoms of emotional disorders (measured at T2,T3, T4, and T5), and a time-invariant factor for symptoms of behavioral disorders (measured at T2,T3, T4, and T5). The latent factor is a time-invariant factor that correlates with the initial measures (sleep duration T2 and symptoms of psychiatric disorders T2) and loads on the respective measures, e.g., on sleep duration (T3-T5). In random effects models, the correlations between initial measures (sleep duration T2) and the time-invariant factor are fixed to zero, whereas in fixed effects models these correlations are freely estimated. In a hybrid model, the initial measures (T2) shown to be uncorrelated with the time-invariant factor are fixed, whereas those who are associated with the latent factor are freely estimated. Time-invariant factor part (a) and fixed/random(b); (c) cross-lagged paths.

© 2019 Ranum BM et al. *JAMA Network Open.*

eEquation. The Regression Equation for the Dynamic Panel Model

$$Y_{it} = \alpha_t + \mu_i + \boldsymbol{\beta} \boldsymbol{X}_{it} + \varepsilon_{it}$$

Note: Y_{it} is the value of the dependent variable for the participant *i* at time *t;* α captures all relevant differences between participants that vary across time and not accounted for by the other independent variables in the model; μ_i captures all relevant differences between participants that are stable over time and not accounted for by the other independent variables in the model; β is a row vector of regression coefficients; X is a column vector of the causes of Y; ε is a random disturbance (error term).[3]

© 2019 Ranum BM et al. *JAMA Network Open.*

eTable 1. Participation Patterns for Children With Valid Sleep Measures

Freqency	Percent	Cumulative percent	Pattern
381	47.68	47.68	1111
69	8.64	56.32	1...
69	8.64	64.96	111.
52	6.51	71.46	.111
50	6.26	77.72	1.11
43	5.38	83.10	11..
40	5.01	88.11	11.1
23	2.88	90.99	1.1.
19	2.38	93.37	.11.
15	1.88	95.24	..11
11	1.38	96.62	.1..
10	1.25	97.87	1..1
8	1.00	98.87	..1.
6	0.75	99.62	...1
3	0.38	100.00	.1.1
799	100.00		

Note: the pattern of "1" and "." denotes the pattern of participation (1) vs nonparticipation (.). The firs symbol represents the first wave of accelerometer measurement; the second symbol represent second wave of accelerometer measurement etc.

© 2019 Ranum BM et al. *JAMA Network Open.*

**eTable 2.** Missing Values on Study Variables		
	Missing	Total
Sleep duration age 6	0	687
Sleep duration age 8	0	619
Sleep duration age 10	0	618
Sleep duration age 12	0	558
Symptoms of emotional disorders age 6	5	687
Symptoms of emotional disorders age 8	5	687
Symptoms of emotional disorders age 10	4	619
Symptoms of emotional disorders age 12	4	619
Symptoms of behavioral disorders age 6	1	618
Symptoms of behavioral disorders age 8	1	618
Symptoms of behavioral disorders age 10	0	558
Symptoms of behavioral disorders age 12	0	558
Weight variable age 6	4	687
Weight variable age 8	3	619
Weight variable age 10	2	618
Weight variable age 12	3	558
Gender age 6	1	687
Gender age 8	0	619
Gender age 10	1	618
Gender age 12	1	558
Note: "Total" refers to number of participants with valid sleep measure at each wave.		

© 2019 Ranum BM et al. *JAMA Network Open.*

eTable 3. Results of Model Fitting Procedure

	X^2	df	P-value	RMSEA[a] (95% CI)	SRMR[b]	CFI[c]	TLI[d]
M1: Baseline model	1517.02	63	<.001				
M2: Fixed effects model	14.80	12	.25	.017 (.000, .042)	.019	.998	.990
**M3: Hybrid model**	**20.02**	**16**	**.22**	**.018 (.000, .039)**	**.021**	**.997**	**.989**

Note: Preferred model in bold. X^2 Chi-square value; df Degrees of freedom; P-value of the Chi-square test of model fit; [a] Root mean square error of approximation; [b] Standardized root mean square residual; [c] Comparative fit index; [d] Tucker Lewis Index.

© 2019 Ranum BM et al. *JAMA Network Open.*

eTable 4. All Unstandardized Regression Coefficients (B) From the Hybrid Model Testing Longitudinal Associations Between Sleep Duration and Symptoms of Psychiatric Disorders

	B (95% CI)	p-value
Sleep duration age 8 regressed on sleep duration age 6	.07 (÷.05, .19)	.25
Sleep duration age 10 regressed on sleep duraiton age 8	.11 (÷.05, .27)	.19
Sleep duration age 12 regressed on sleep duration age 10	.11 (÷.05, .28)	.19
Sleep duration age 8 regressed on symptoms of emotional disorders age 6	.00 (÷.03, .02)	.73
Sleep duration age 10 regressed on symptoms of emotional disorders age 8	÷.02 (÷.04, .01)	.14
Sleep duration age 12 regressed on symptoms of emotional disorders age 10	.00 (÷.03, .02)	.81
Sleep duration age 8 regressed on symptoms of behavioral disorders age 6	.00 (÷.02, .02)	.90
Sleep duration age 10 regressed on symptoms of behavioral disorders age 8	.01 (÷.01, .02)	.38
Sleep duration age 12 regressed on symptoms of behavioral disorders age 10	.00 (÷.01, 02)	.58
Symptoms of emotional disorders age 8 regressed on sleep duration age 6	÷.44 (÷.80, ÷.08)	.02
Symptoms of emotional disorders age 10 regressed on sleep duration age 8	÷.47 (÷.83, ÷.11)	.01
Symptoms of emotional disorders age 12 regressed on sleep duration age 10	.05 (÷.40, .50)	.83
Symptoms of emotional disorders age 8 regressed on symptoms of emotional disorders age 6	.02 (÷.08, .12)	.71
Symptoms of emotional disorders age 10 regressed on symptoms of emotional disorders age 8	÷.07 (÷.25, .10)	.41
Symptoms of emotional disorders age 12 regressed on symptoms of emotional disorders age 10	.22 (.11, .33)	<.001
Symptoms of emotional disorders age 8 regressed on symptoms of behavioral disorders age 6	.08 (÷.02, .19)	.13
Symptoms of emotional disorders age 10 regressed on symptoms of behavioral disorders age 8	.11 (÷.03, .25)	.12
Symptoms of emotional disorders age 12 regressed on symptoms of behavioral disorders age 10	.05 (÷.07, .17)	.83
Symptoms of behavioral disorders age 8 regressed on sleep duration age 6	÷.35 (÷.73, .05)	.08
Symptoms of behavioral disorders age 10 regressed on sleep duration age 8	÷.49 (÷.90, .÷.07)	.02
Symptoms of behavioral disorders age 12 regressed on sleep duration age 10	÷.33 (÷.69, .04)	.08
Symptoms of behavioral disorders age 8 regressed on symptoms of emotional disorders age 6	÷.03 (÷.14, .09)	.66
Symptoms of behavioral disorders age 10 regressed on symptoms of emotional disorders age 8	÷.05 (÷.20, .10)	.53
Symptoms of behavioral disorders age 12 regressed on symptoms of emotional disorders age 10	.09 (÷.04, .21)	.17
Symptoms of behavioral disorders age 8 regressed on symptoms of behavioral disorders age 6	.31 (.16, .46)	<.001
Symptoms of behavioral disorders age 10 regressed on symptoms of behavioral disorders age 8	.30 (.11, .48)	.001
Symptoms of behavioral disorders age 12 regressed on symptoms of behavioral disorders age 10	.20 (÷.01, .41)	.06

© 2019 Ranum BM et al. *JAMA Network Open.*

eAppendix 1. Mplus Code for a Hybrid, Fixed, and Random Effects Dynamic Panel Model Testing Longitudinal Associations Between Sleep Duration and Symptoms of Psychiatric Disorders

```
weight is w;
usevariables are sd2-sd5 ed2-ed5 bd2-bd5;

ANALYSIS:
ESTIMATOR=MLR;

Model:
a by sd3@1 sd4@1 sd5@1;
b by ed3@1 ed4@1 ed5@1;
c by bd3@1 bd4@1 bd5@1;

a with sd2 ed2 bd2;
b with sd2@0 ed2 bd2;
c with sd2@0 ed2 bd2;
b with a@0;
c with a@0;

ed5 on ed4 bd4 sd4;
bd5 on ed4 bd4 sd4;
sd5 on ed4 bd4 sd4;

ed4 on ed3 bd3 sd3;
bd4 on ed3 bd3 sd3;
sd4 on ed3 bd3 sd3;

ed3 on ed2 bd2 sd2;
bd3 on ed2 bd2 sd2;
sd3 on ed2 bd2 sd2;

sd5 with bd5 ed5;
sd4 with bd4 ed4;
sd3 with bd3 ed3;
sd2 with bd2 ed2;

ed5 with bd5;
ed4 with bd4;
ed3 with bd3;
ed2 with bd2;

output: stdyx samp cinterval;
```

Note: sd = sleep duration ed = symptoms of emotional disorders, bd = symptoms of behavioral disorders. The number after each letter code denotes the data collection wave, e.g. sd2 = sleep duration at wave 2 (i.e. 6 years of age). w is variable identifying the number of children in each stratum in the population.

© 2019 Ranum BM et al. *JAMA Network Open.*

eAppendix 2. The Dynamic Panel Model (Random, Fixed, and Hybrid Effects Models): General Issues

Time-invariant and time-varying variables. Variables may be considered to be of two types: (1) Time-invariant factors such as genetics and gender, who do not change over time and (2) time-varying variables that do change over time (e.g. life-events, friendships, schooling). For further details, see references.[1,2]

A fixed effects model implies that the effects of time-varying predictors (e.g., sleep duration) are adjusted for the confounding effect of time-invariant factors. In the present work, that what causes stability of sleep duration and symptoms of psychiatric disorders is partialled out by correlating initial measures of sleep duration and initial measures of psychiatric symptoms (i.e., the predictors on T2) with the time-invariant factor. This approach is equivalent to the method of adjusting for confounders in ordinary regression.

In contrast to fixed effects regression, who only utilizes within-person variation (i.e., participants serve as their own control), *random effects models* use both within- and between-person information. Such models have therefore more statistical power. However, in a random effects model predictors are assumed to be uncorrelated with the time-invariant factor(s). Thus, random effects models are appropriate when predictors are proven to be uncorrelated with the time-invariant factor(s) and setting these correlations to 0 does not deteriorate the fit of the model. In other words, there is no need to adjust for the potential confounding effect of time-invariant common causes between sleep duration and symptoms of psychiatric disorders if there is no correlation between the time-invariant factor and the predictors.

Hybrid models consisting of both fixed and random effects are also possible.[3] In these models, some predictors are set to correlate with the time-invariant factor(s), whereas other predictors are not. Hybrid models retain the fixed effects advantage of adjusting for time-invariant factors (when these may influence the results) while being more parsimonious and more statistically powerful than pure fixed effects models.[3,4]

© 2019 Ranum BM et al. *JAMA Network Open.*

eAppendix 3. Fitting of the Models

The goodness-of-fit statistics from fitting fixed, random and hybrid effects models are displayed in Table S1. RMSEA, CFI and TLI is assorted tests concerning how well the hypothesized model fit the data. Root Mean Square Error of Approximation (RMSEA) is a parsimony-adjusted index. Values closer to zero represents a good fit. The comparative fit index (CFI) analyzes the model fit by examining the discrepancy between the data and the hypothesized model, while adjusting for the issues of sample size inherent in the chi-squared test of model fit, and the normed fit index. A Tucker Lewis index (TLI) of .95, indicates the model of interest improves the fit by 95% relative to the null model. The As can be seen, a hybrid model (M3) did not fit not fit the data significantly worse than a fixed effects model (M2). Because hybrid models retain the fixed effects advantage of adjusting for time-invariant factors (when these may influence the results) while being more parsimonious and more statistically powerful than pure fixed effects models,[3,4] these were the preferred models.

© 2019 Ranum BM et al. *JAMA Network Open.*

eReferences

1. Wichstrøm L, Belsky J, Steinsbekk S. Homotypic and heterotypic continuity of symptoms of psychiatric disorders from age 4 to 10 years: a dynamic panel model. *J Child Psychol Psychiatry.* 2017:58(11):1239-1247.
2. Wichstrøm L, Penelo E, Rensvik Viddal K, de la Osa N, Ezpeleta L. Explaining the relationship between temperament and symptoms of psychiatric disorders from preschool to middle childhood: hybrid fixed and random effects models of Norwegian and Spanish children. *J Child Psychol Psyc.* 2018;59(3):285-295.
3. Firebaugh G, Warner C, Massoglia M. Fixed Effects, Random Effects, and Hybrid Models for Causal Analysis. In: Morgan SL, ed. *Handbook of Causal Analysis for Social Research.* Dordrecht: Springer Science+Business Media; 2013:113-132.
4. Allison PD. *Fixed effects regression models.* Vol 160: SAGE publications; 2009.

© 2019 Ranum BM et al. *JAMA Network Open.*
